# Consistency and Variability of the Human Milk Oligosaccharide Profile in Repeat Pregnancies

**DOI:** 10.3390/nu16050643

**Published:** 2024-02-25

**Authors:** Simone Renwick, Kamand Rahimi, Kristija Sejane, Kerri Bertrand, Christina Chambers, Lars Bode

**Affiliations:** 1 Department of Pediatrics, School of Medicine, University of California San Diego, La Jolla, CA 92093, USA; srenwick@health.ucsd.edu (S.R.); karahimi@health.ucsd.edu (K.R.); ksejane@health.ucsd.edu (K.S.); kdutcher@health.ucsd.edu (K.B.); chchambers@health.ucsd.edu (C.C.); 2 Mother-Milk-Infant Center of Research Excellence, University of California San Diego, La Jolla, CA 92093, USA; 3 UC San Diego Mommy’s Milk Human Milk Research Biorepository, University of California San Diego, La Jolla, CA 92093, USA; 4 Herbert Wertheim School of Public Health and Human Longevity Science, University of California San Diego, La Jolla, CA 92093, USA; 5 Human Milk Institute, University of California San Diego, La Jolla, CA 92093, USA

**Keywords:** human milk, human milk oligosaccharides, lactation, parity, HPLC

## Abstract

Human milk oligosaccharides (HMOs) are a set of complex carbohydrates and the third largest solid component of human milk, after lactose and lipids. To date, over 150 HMOs have been identified and the diversity of structures produced by lactating women is influenced by maternal genetics as well as other maternal, infant, and environmental factors. While the concentrations of individual HMOs have been shown to vary between individuals and throughout the course of lactation, the variability of HMO concentration profiles following different pregnancies occurring in the same woman is presently unknown. As such, the objective of this study was to compare HMO concentrations in human milk samples provided by the same women (*n* = 34) following repeat pregnancies. We leveraged existing human milk samples and metadata from the UC San Diego Human Milk Research Biorepository (HMB) and measured the concentrations of the 19 most abundant HMOs using high-performance liquid chromatography with fluorescence detection (HPLC-FL). By assessing dissimilarities in HMO concentration profiles, as well as concentration trends in individual structures between pregnancies of each participant, we discovered that HMO profiles largely follow a highly personalized and predictable trajectory following different pregnancies irrespective of non-genetic influences. In conclusion, this is the first study to assess the interactions between parity and time following delivery on variations in HMO compositions.

## 1. Introduction

The optimal nutrient source for healthy-term infants during their first six months of life is human milk owing to its diversity of macro- and micronutrients as well as bioactive compounds. These nutrients vary in concentration throughout the period of lactation, presumably reflecting the evolving nutritional demands of the growing infant. Following lactose and lipids, human milk oligosaccharides (HMOs) are the third most abundant class of molecules found in human milk, ranging from 5 to more than 15 g/L in mature milk [1]. To date, over 150 distinct HMO structures, comprising differing combinations of glucose, galactose, N-acetylglucosamine, fucose, and sialic acid, have been identified. The assembly of HMOs involves the elongation of a lactose core with lacto-N-biose or N-acetyllactosamine units. These core backbone structures can be further modified by fucose and/or sialic acid in different linkages [1]. 

HMO composition varies greatly between individuals, which is mostly determined by genetics and vastly influenced by polymorphisms in the Secretor (Se) and Lewis (Le) blood group genes that encode the α1-2-fucoslyltransferase (FUT2) and α1-3/4-fucosyltransferase (FUT3) enzymes, respectively [2]. As such, women are differentiated into four milk groups with the following prevalence in the Caucasian population: 69% Se+Le+, 20% Se−Le+, 9% Se+Le−, and 1% Se−Le− [3]. Furthermore, Se+ individuals (referred to as secretors) constitute 50–80% of the Chinese population, 70% of the Vietnamese population, 83–100% of the Taiwanese population, almost 100% of Mexicans and Peruvians, and 65–80% of people in parts of Africa [4]. Moreover, factors other than genetics may influence an individual’s HMO profile, including their age, pre-pregnancy body-mass index (BMI), delivery mode, lactation stage, breastfeeding exclusivity, and infant sex [5,6,7,8]. However, how consistent or variable HMO compositions are within the same individual over repeat pregnancies remains unknown. In addition, HMO composition changes over the course of lactation where most HMOs decline in concentration [7,9], but some HMOs, such as 3-fucosyllactose (3FL), 3′sialylactose (3′SL), and lacto-N-fucopentaose (LNFP) 3, increase in concentration [7,8,10]. Whether these same changes over the course of lactation occur in the same individual over repeat pregnancies is also unknown.

To address these knowledge gaps, we leveraged existing human milk samples and metadata from the UC San Diego Mommy’s Milk Human Milk Research Biorepository (HMB) to study the impact of various maternal–infant characteristics, including lactation stage, total time in between milk samples from repeat pregnancies, infant gestational age, and infant sex, on the variability in HMO concentrations in the human milk of participants across multiple pregnancies.

## 2. Materials and Methods

### 2.1. Participants and Sample Collection

Human milk samples provided to the Mommy’s Milk HMB between 2014 and 2022 by the same study participant following different pregnancies were selected for analysis. Participants comprised lactating women 18 years and older who resided in the United States. Samples were collected following the HMB protocol [11]. At the time of sample collection, participants provided written informed consent for use of their samples for research. Participants were also interviewed about their sociodemographic characteristics, medical history, diet, and lifestyle. To obtain representative samples, participants were asked to express 50 mL of milk containing both foremilk and hindmilk. Samples were collected at participants’ homes, community centers, or at UC San Diego. Samples collected at UC San Diego were kept on ice for up to 3 h before aliquoting and storage at the HMB at −80 °C. Samples collected off-site were stored at 4 °C for up to 3 h before pickup or shipped overnight on ice to HMB for aliquoting and storage at −80 °C. The study was approved by the institutional review board (IRB) at UC San Diego under Project #130658.

### 2.2. Quantification of HMOs

HMOs were measured using high-performance liquid chromatography (HPLC) as previously described [12]. Briefly, the non-HMO oligosaccharide D-maltose monohydrate (Sigma-Aldrich, St. Louis, MO, USA) was added to the samples selected for this study as an internal standard prior to sample processing. Samples were lyophilized using a speed-vacuum before glycans were labeled with 2-aminobenzamide (2AB) at 65 °C for 2 h. 2AB-glycans were separated by HPLC with fluorescent detection (HPLC-FL) (Dionex Ultimate 3000, Thermo Fisher Scientific, Waltham, MA USA) on a TSKgel amide-80 column (15 cm length, 2 mm inner diameter, 3 μm particle size; Tosoh Bioscience, King of Prussia, PA, USA). Annotation of peaks was based on standard retention times. The absolute concentrations of the following HMOs were calculated based on the area under the curve for the internal standard maltose and the reference standards of each of the individual HMOs: 2′-fucosyllactose (2′FL), 3 FL, 3′SL, 6-sialyllactose (6′SL), difucosyllactose (DFLac), difucosyllacto-N-hexaose (DFLNH), difucosyllacto-N-tetrose (DFLNT), disialyllacto-N-hexaose (DSLNH), disialyllacto-N-tetraose (DSLNT), fucodisialyllacto-N-hexaose (FDSLNH), fucosyllacto-N-hexaose (FLNH), LNFP1, LNFP2, LNFP3, lacto-N-hexaose (LNH), lacto-N-tetrose (LNT), lacto-N-neo-tetrose (LNnT), sialyllacto-N-tetraose (LST) b, and LSTc. HMO-bound sialic acid (Sia) and HMO-bound fucose were calculated on a molar basis (nmol/mL). 

### 2.3. Statistical Analysis

Statistical analysis was conducted using R (version 4.1.2) using code hosted at https://github.com/SimoneRenwick/Nutrients2024 (accessed on 16 February 2024). To visualize compositional variation among the milk samples, a Principal coordinates analysis (PCoA) of Bray–Curtis dissimilarity was generated. Bray–Curtis dissimilarity was calculated between first and subsequent milk samples from the same participant, as well as between random participants with the same secretor status. These measures were then projected against the difference in the time of lactation at which the first and subsequent samples were provided (t2 − t1). The strength of the correlation between dissimilarity measures and absolute difference in time of lactation was quantified by Spearman rank correlation. Dissimilarity values, difference in lactation time, intervals between sample collections, differences in the gestational age at birth of the consecutive infants, and percentage changes in individual HMO concentrations from first to subsequent delivery for each participant were assessed for normality using the Shapiro–Wilk test. All variables that significantly deviated from the normal distribution were logarithmically transformed and again evaluated for normality. Subsequently, a multivariate regression was used to evaluate the impact of maternal–infant factors on both the Bray–Curtis dissimilarity and changes in concentration of individual HMOs. In the case of FDSLNH, which could not be normalized, an alternative Kendall–Theil Sen Siegel nonparametric linear regression was used to assess the influence of differences in lactation stage, gestational age at birth of the consecutive infants, and time of sample collection on changes in concentration. The nonparametric Kruskal–Wallis test was used to evaluate the impact of secretor status and infant sex on change in FDSLNH concentration. 

## 3. Results

### 3.1. Participants and HMO Profiles

**Figure 1:**
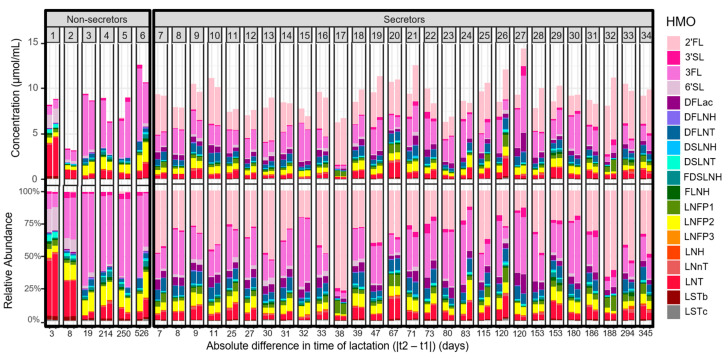
Concentrations (μmol/mL) and relative abundance (%) of 19 HMOs in samples from participants (*n* = 34) who provided human milk following two different pregnancies. First and subsequent samples were paired for each participant. Participants are numbered 1–34 and grouped by secretor status. Participants are ordered along the *x*-axis by increasing absolute difference in the times of lactation when samples were provided.

Human milk samples provided by 34 participants following two pregnancies (samples = 68) were selected for analysis. Aside from five participants, all provided milk samples following the births of their first and second children. Exceptions included one participant who contributed samples following the births of their first and third children; two participants who contributed samples following their first delivery of twins, and then again after the birth of a single child; one participant who contributed samples following their first delivery of a single child and then again after twins; and one participant who provided samples following two deliveries of twins. All except nine infants were born full-term (37–42 weeks gestational age). Exceptions included six infants born moderate to late preterm (32–36 weeks gestational age) and one extremely preterm infant (25 weeks gestational age). Time between sample collections ranged from 416 to 1616 days (1.14 to 4.43 years), with the average being 892 days (2.44 years). From provision of the first sample to that of the second sample, the difference in lactation stage (days postpartum for each of the two pregnancies) ranged from −526 days (−1.44 years, first sample collected later in lactation than second sample) to 250 days (0.68 years, first sample collected earlier in lactation than second sample) (Supplementary Figure S1). Excluding a participant who delivered mixed-sex twins, 13 women contributed samples after delivering infants of different sexes, while 11 women delivered only males and 9 women delivered only females. The HMO abundance profiles for paired milk samples provided by each participant are displayed in Figure 1. Based on the abundance of α1-2-fucoslylated HMOs in their milk, 82.4% *(n =* 28) participants were classified as secretors, while 17.6% *(n =* 6) participants were classified as non-secretors. 

### 3.2. Bray–Curtis Dissimilarity in HMO Profiles across Different Deliveries

**Figure 2:**
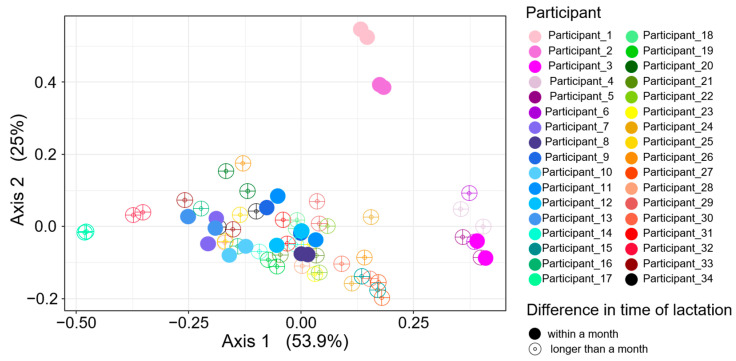
Principal coordinate analysis (PCoA) based on Bray–Curtis dissimilarity measures of the HMO composition of milk samples (*n* = 68) provided following different pregnancies. Each circle represents the HMO composition of a given sample. Circles with the same color represent samples from the same subject but different pregnancies. Closed circles denote samples provided within a month postpartum, while open circles denote longer than a month.

The Bray–Curtis dissimilarity statistic was used to assess the homogeneity of the HMO profiles among the milk samples. Initially, dissimilarity among all samples was calculated and visualized using a PCoA plot (Figure 2). Samples collected from the same woman demonstrated pronounced ordination proximity, signifying a large degree of similarity in their HMO profiles even though they were provided following different pregnancies. Notably, samples frequently formed even tighter clusters when provided at comparable postpartum time points, typically within a month’s difference.

**Figure 3:**
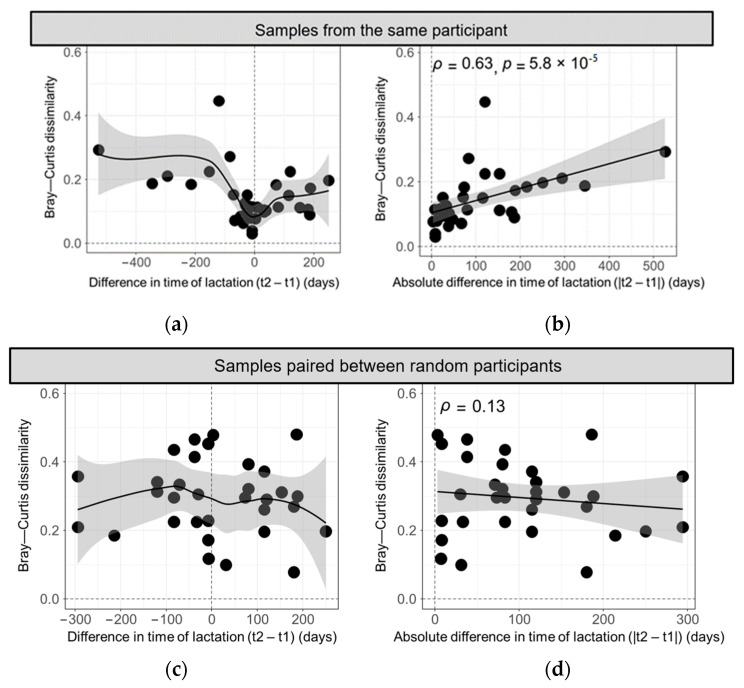
Bray–Curtis dissimilarity metrics comparing the HMO profiles of milk samples provided by (**a**,**b**) the same participant and (**c**,**d**) randomized participants with the same secretor status. Dissimilarity metrics are graphed in the context of (**a**,**c**) the difference in the time of lactation following birth (t2 − t1) and (**b**,**d**) the absolute difference in the time of lactation following birth (|t2 − t1|). Each circle represents a single participant. Black trendlines were fitted with either (**a**,**c**) local polynomial regression (locally weighted scatterplot smoothing (loess)) or (**b**,**d**) a generalized linear model. The grey areas around the trendlines represent the 95% confidence interval. The strength of the correlation between Bray–Curtis dissimilarity metrics and absolute difference in lactation time was evaluated using Spearman rank correlation coefficient, ρ.

Next, the Bray–Curtis dissimilarity statistic was calculated between pairs of samples in order to further evaluate how participant and stage in lactation impacted the overall similarity in the HMO profiles of the samples. First, samples originating from the same participant were compared and plotted against the difference in lactation time. Unsurprisingly, samples provided at similar times postpartum exhibited a considerable likeness as they clustered close to the origin, whereas larger variations were observed in samples provided further apart in postpartum time (Figure 3a). Moreover, a significant moderate correlation emerged between the dissimilarity of the paired milk samples of each participant and the absolute difference in time of lactation (Spearman’s ρ = 0.63, *p* < 0.0001) (Figure 3b). Hence, participant HMO profiles increasingly diverged when provided at different lactation stages. However, when samples were paired between random participants sharing the same secretor status, their Bray–Curtis dissimilarity measures no longer clustered near the origin (Figure 3c), nor did they correlate with the absolute difference in time of lactation (Figure 3d). Consequently, HMO profiles are most similar between samples obtained from the same participant and when collected at similar times postpartum. 

### 3.3. Influence of Parity and Lactation Time on Individual HMOs

**Figure 4:**
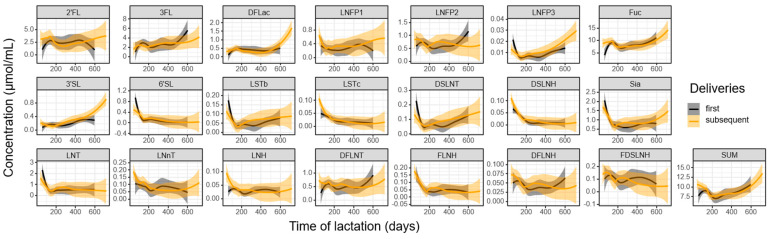
Change in the concentration (μmol/mL) of 19 HMOs, as well as HMO-bound sialic acid (Sia), HMO-bound fucose (Fuc), and total quantified HMOs (SUM), over the course of lactation following first and subsequent deliveries. Trendlines were fitted with a local polynomial regression (locally weighted scatterplot smoothing (loess)). The grey and transparent orange areas around the trendlines represent the 95% confidence interval of the first and subsequent deliveries, respectively.

We next considered the influence of parity and lactation time on the concentration of individual HMOs, as well as HMO-bound Sia and Fuc. Upon parsing samples by first or subsequent delivery, several trends emerged (Figure 4). Throughout the lactation period, numerous oligosaccharides declined in concentration, with the most precipitous decrease occurring in the first 100 days. Notable examples of this trend included 6′SL, LSTc, and DSLNH. In contrast, certain structures displayed an initial rapid decline followed by stabilization for the remainder of lactation. This pattern was observed in core HMO structures such as LNT, LNnT, and LNH. Conversely, some oligosaccharides exhibited an increase in concentration during the later stages of lactation, as evident in the trajectory of DFLac, LNFP3, and DFLNT. Notably, 3FL and 3′SL deviated from the trends of the other structures as they demonstrated an increase in concentration over time. These general trends were largely consistent following both first and subsequent deliveries. 

**Figure 5:**
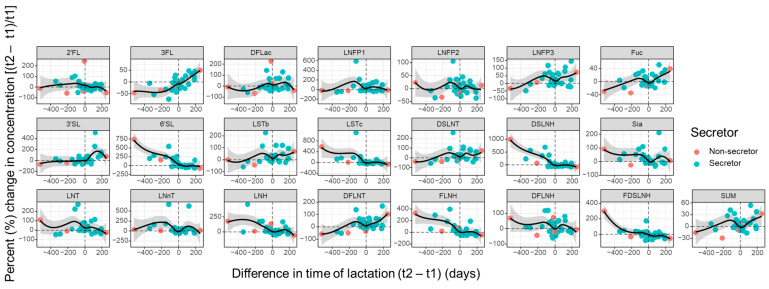
Percentage (%) change in the concentration of 19 HMOs, as well as HMO-bound sialic acid (Sia), HMO-bound fucose (Fuc), and total quantified HMOs (SUM), from first to subsequent delivery ((t2 − t2)/t1), compared to the difference in time of lactation when samples were collected (t2 − t1). Each circle represents a single participant and is colored by the participant’s secretor status. Black trendlines were fitted with a local polynomial regression (locally weighted scatterplot smoothing (loess)). The grey area around the trendlines represents the 95% confidence interval.

After viewing trends in HMO concentrations at the population level for different pregnancies, we next sought to understand the changes occurring at the individual level. As such, we compared the individual oligosaccharides of each participant from their first to their subsequent pregnancy sample collection. When the percent change was evaluated in the context of the differences in lactation stage when the samples were collected, trends seen at the population level were preserved (Figure 5). For example, the concentrations of 3FL, 3′SL, and LNFP3 exhibited an increase in later samples, while the concentrations of 6′SL, LSTc, DSLNH, LHN, FLNH, and FDSLNH were higher in earlier samples. When samples were provided at similar times postpartum (close to 0 days difference in time of lactation), the variation in the concentrations of individual structures were minimal, nearing 0% change. This underscores the consistency in the trajectory of HMO profiles within the same participant and across different births.

### 3.4. Influence of Maternal and Infant Factors on HMO Profiles

**Figure 6:**
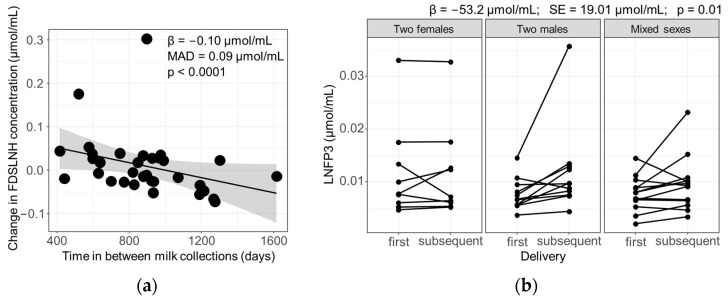
HMOs that were significantly affected by maternal–infant characteristics. (**a**) Effect on the concentration of FDSLNH was determined using Kendall–Theil Sen Siegel nonparametric linear regression. The trendline was fitted with a local polynomial regression (locally weighted scatterplot smoothing (loess)). The grey area around the trendline represents the 95% confidence interval. The beta (β) coefficient, median absolute deviation (MAD), and *p*-value (*p*) are denoted in the figure. (**b**) Concentration of LNFP3 in milk samples provided following first and subsequent deliveries stratified by the combination of infant sex. Each point represents a milk sample. Lines connect the two samples provided by each participant. Effect on the concentration of LNFP3 was determined using a multivariate regression. The β coefficient, standard error (SE), and *p* of the regression are denoted above the figure.

The influence of maternal–infant factors, including lactation stage, time in between sample collections, secretor status, infant gestational age, and combination of infant sex, on the consistency of the HMO profiles of participants following different pregnancies were analyzed using a multivariate regression. The findings revealed that the Bray–Curtis dissimilarity measures between a participant’s samples remained unaffected by the time between sample collections, the participant’s secretor status, the difference in the gestational age at birth of the consecutive infants, and the sex of the infants. However, upon examination of the individual oligosaccharides, a significant decline in the concentration of FDSLNH was evident when samples were provided further apart in time (β = −0.10 μmol/L, median absolute deviation = 0.09 μmol/L, *p* < 0.0001) (Figure 6a). In addition, the infants’ sex also influenced the concentration of LNFP3 as mothers who gave birth to two males produced a higher concentration following their subsequent delivery, in comparison to mothers of two females or mixed-sex offspring (β = 53.2 μmol/L, standard error = 19.01 μmol/L, *p* = 0.01) (Figure 6b). Among participants with mixed-sex infants, the sequence of birth did not significantly alter the LNFP3 concentrations. 

## 4. Discussion

In the present study, we investigated the influence of various parent–child characteristics on the consistency of HMO profiles following two different pregnancies in the same individual. Overall, the HMO composition was observed to be highly conserved across different pregnancies, suggesting that maternal genetics in general and the expression of fucosyltransferases in particular are the main drivers of HMO composition, which persists across multiple pregnancies. We further noted that the concentrations of expressed HMOs followed a predictable personalized trajectory over the course of lactation as samples from a given participant were most similar when provided at comparable time points postpartum. Changes in the HMOs produced over time likely reflected the evolving needs of the infant at various developmental stages. HMOs fulfill a multitude of critical roles, encompassing their function as prebiotic substrates for beneficial gut microbes [13,14,15], their capacity to reduce the risk of infection by serving as soluble decoy receptors [16,17], and their ability to modulate the immune system through binding to cell surface receptors on both epithelial and immune cells [18]. 

Previous studies have documented a decline in the total concentration of HMOs in all milk groups over the course of lactation, with the most rapid reduction observed within the first three months [7,9,19]. Most individual HMOs are generally reported to decrease in concentration throughout the lactation period, with several notable exceptions which have been shown to increase in concentration: 3FL [8,10,19,20], 3′SL [7,9,10,19], DFLac [10,19], and LNFP3 [10]. These findings align with our study and support the notion of distinct roles for individual HMO structures during the various stages of infant development. Oligosaccharides that present in higher concentrations during early lactation may serve specific functions in early growth and immunity, while HMOs that stabilize or increase over time may function by supporting the maturation of the intestine and gut microbiota at later stages. Following an initial decline, we observed a consistent concentration of core structures, including LNT and LNnT, which are known substrates of *Bifidobacterium* species, dominant taxa in the healthy, breastfed infant gut [21]. However, further research is required to elucidate the biological significance of the observed concentration trajectories. 

To date, several longitudinal and cross-sectional studies have sought to investigate the impact of maternal and infant factors on the HMO composition of milk. Parameters such as maternal age, parity, pre-pregnancy BMI, allergies, dietary habits, mode of delivery, breastfeeding exclusivity, socioeconomic status, and pet ownership, as well as infant sex, birth weight, gestational age, growth measures, and body composition, have been explored [5,6,7,8,10,19,20,22]. However, to our knowledge, our study is the first to compare participants across different pregnancies, thereby facilitating an investigation into the impact of maternal–infant characteristics on intra-individual variation in oligosaccharide concentrations. Interestingly, we observed a reduction in the concentration of FDSLNH with an increasing interval between deliveries. This observation suggests a negative correlation between FDSLNH concentrations and maternal age and/or parity. Previously, Wang et al. [8] noted a similar negative association between parity and the concentration of several long-chain HMOs, including difucosyl-para-lacto-N-hexaose I (FDLNH1), a possible precursor in the synthesis of FDSLNH. However, our observations contrast other studies that have reported positive correlations between parity and FDSLNH [22], LNT, and LNnT [6,7,20], negative correlations with 3FL [6,20], DFLac, LNFP1, and LNFP4 [8], or no discernible associations [23]. However, none of these studies compared HMO composition by parity within the same individuals, i.e., consecutive pregnancies.

While previous studies have reported associations between infant sex and the macronutrient content [24,25,26] and hormone levels [27] of human milk, there is limited research examining associations of infant sex with maternal HMO composition. A prior study by Tonon et al. [20] indicated that daughters of Se+Le- mothers were associated with higher concentrations of 2′FL in their mothers’ milk while sons were associated with higher concentrations of LNH, LNT, and LNnT. Furthermore, Wang et al. [8] reported that secretor mothers of males produced higher concentrations of LNH, Lacto-N-neohexaose (LNnH), LNFP1, FLNH1&3, and DFLNH-a&c compared to the mothers of females. However, Siziba et al. [19] reported no significant effects of infant sex on HMO composition. In the present study, we observed that mothers who delivered two males produced substantially higher concentrations of LNFP3 when feeding their subsequent son as opposed to mothers of two females or those with mixed-sex offspring in consecutive pregnancies. Notably, this increase was only observed among consecutive sons as the birth of a son subsequent to a daughter did not alter LNFP3 concentrations. It is imperative to acknowledge that an array of factors, both defined and undefined, may contribute to changes in HMO concentrations and, as such, correlations should be interpreted with caution. 

Studying the composition of human milk from the same individual but from consecutive pregnancies comes with inherent challenges. Participant retention from one pregnancy to the next is likely low and timing between different pregnancies is unpredictable, thereby influencing the overall duration of such a study. Here, we leveraged existing human milk samples and metadata from the UC San Diego Mommy’s Milk HMB that has employed an open cohort model recruiting lactating participants across the U.S. and Canada for over nine years. Due to their large recruitment numbers (*n* > 3100), the HMB has also included participants who provided milk samples from two or more different pregnancies, allowing for a comprehensive exploration of factors that affect intra-individual variation in HMO composition across pregnancies within the same individuals. Deviating from previous studies that utilized analytical platforms that exclusively yielded relative HMO abundances, the present study quantified absolute HMO concentrations. This approach facilitated the calculation of changes in both total and individual HMO concentrations, mitigating challenges associated with the interdependency observed in relative abundance profiles. An additional strength of this study lies in the novel application of the Bray–Curtis dissimilarity statistic borrowed from the field of ecology to assess compositional variation. 

## 5. Conclusions

In summary, by leveraging access to human milk samples collected through the HMB, our study evaluated the influence of maternal and infant characteristics on the consistency and variability of HMO concentration profiles across multiple pregnancies in the same individual. We discovered that HMO profiles largely follow a highly personalized and predictable trajectory following different pregnancies irrespective of non-genetic influences. Additionally, our results supported previous reports of a rapid decline in the concentration of most HMOs within the initial 100 days of lactation, with notable exceptions of 3FL, 3′SL, and DFLac, which displayed an increase over the lactation period. Lastly, concentrations of FDSLNH diminished with increasing time intervals between collections, while concentrations of LNFP3 increased in participants who delivered consecutive male offspring compared to those with two female or mixed-sex offspring.

## Data Availability

The data presented in this article are available on https://github.com/SimoneRenwick/Nutrients2024 (accessed on 16 February 2024).

## Supplementary Materials

The following supporting information can be downloaded at: https://www.mdpi.com/article/10.3390/nu16050643/s1, Figure S1: Time of lactation (days) of samples provided by each participant following their first and subsequent pregnancies.
