# Supplementary material for: Consistency and Variability of the Human Milk Oligosaccharide Profile in Repeat Pregnancies
Source: Nutrients. 2024 Feb 25;16(5):643. doi: 10.3390/nu16050643 (PMC10933908; doi:10.3390/nu16050643)
Supplement: Supplementary file 1 [file nutrients-16-00643-s001.zip › nutrients-2873111-supplementary.pdf]

Consistency and variability of the human milk oligosaccharide profile in repeat pregnancies

Simone Renwick [1,2], Kamand Rahimi [1], Kristija Sejane [1], Kerri Bertrand [1,3,4], Christina Chambers [1,3,4,5], and Lars Bode [1,2,5,*]

1 Department of Pediatrics, School of Medicine, University of California San Diego, La Jolla, CA 92093; srenwick@health.ucsd.edu (S.R.); karahimi@health.ucsd.edu (K.R.); ksejane@health.ucsd.edu (K.S.); kdutcher@health.ucsd.edu (K.B.); chchambers@health.ucsd.edu (C.C.)
2 Mother-Milk-Infant Center of Research Excellence, University of California San Diego, La Jolla, CA 92093
3 Mommy's Milk Human Milk Research Biorepository, San Diego, CA 92123
4 Herbert Wertheim School of Public Health and Human Longevity Science, University of California San Diego, La Jolla, CA 92093
5 Human Milk Institute, University of California San Diego, La Jolla, CA 92093
* Correspondence: lbode@health.ucsd.edu (L.B.)

Supplementary Material

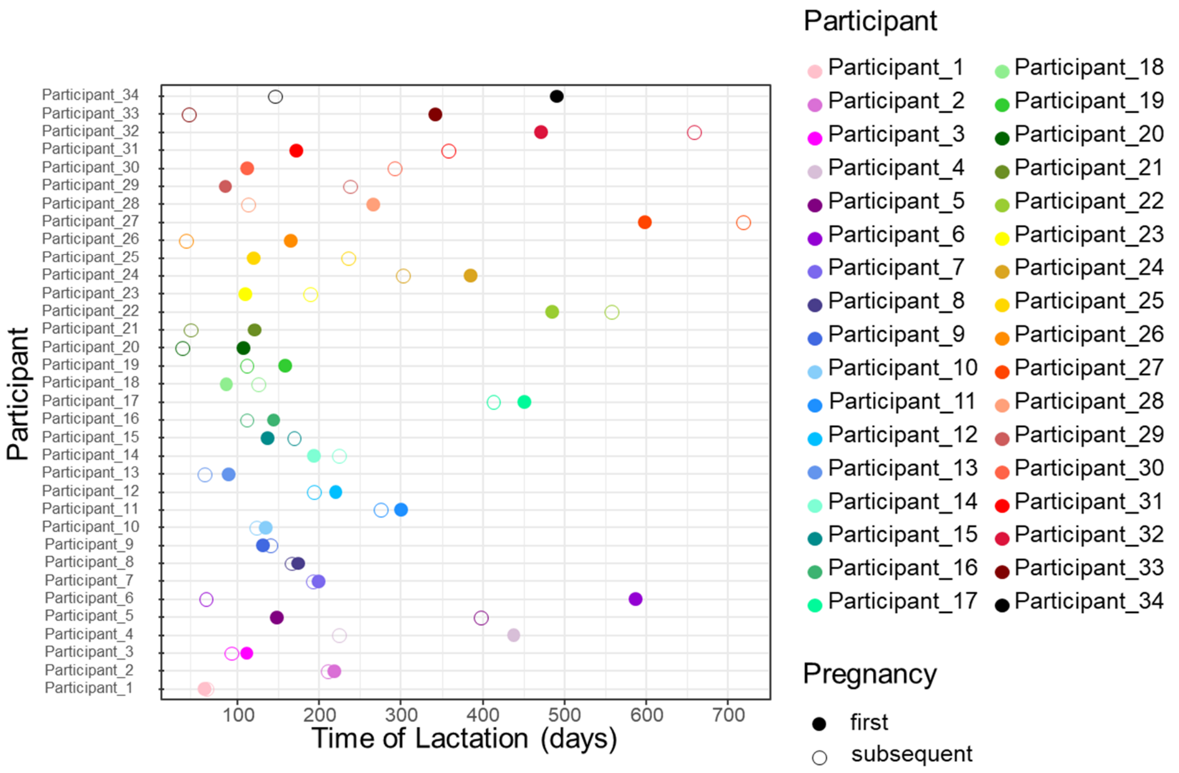

Supplementary Material Figure S1. Time of lactation (days) of samples provided by each participant following their first and subsequent pregnancies.
